# *Bacillus subtilis* ER-08, a multifunctional plant growth-promoting rhizobacterium, promotes the growth of fenugreek (*Trigonella foenum-graecum* L.) plants under salt and drought stress

**DOI:** 10.3389/fmicb.2023.1208743

**Published:** 2023-08-24

**Authors:** Margi Patel, Shaikhul Islam, Fohad Mabood Husain, Virendra Kumar Yadav, Hyun-Kyung Park, Krishna Kumar Yadav, Snehal Bagatharia, Madhvi Joshi, Byong-Hun Jeon, Ashish Patel

**Affiliations:** ^1^Department of Life Sciences, Hemchandracharya North Gujarat University, Patan, Gujarat, India; ^2^Bangladesh Agricultural Research Council, Dhaka, Bangladesh; ^3^Department of Food Science and Nutrition, College of Food and Agriculture Sciences, King Saud University, Riyadh, Saudi Arabia; ^4^Department of Pediatrics, Hanyang University College of Medicine, Seoul, Republic of Korea; ^5^Faculty of Science and Technology, Madhyanchal Professional University, Ratibad, Bhopal, India; ^6^Environmental and Atmospheric Sciences Research Group, Scientific Research Center, Al-Ayen University, Thi-Qar, Nasiriyah, Iraq; ^7^Gujarat State Biotechnology Mission (GSBTM), Gandhinagar, Gujarat, India; ^8^Gujarat Biotechnology Research Centre (GBRC), Gandhinagar, Gujarat, India; ^9^Department of Earth Resources and Environmental Engineering, Hanyang University, Seoul, Republic of Korea

**Keywords:** drought stress, fenugreek, multi-trait endophytic bacteria, rhizobacteria, plant growth augmentation, salt stress

## Abstract

**Introduction:**

Sustainable agriculture and meeting the world's food needs face considerable obstacles from abiotic stresses such as soil salinity and drought. This critical issue was addressed by our current study, which sought to uncover multi-trait bioinoculants from hostile ecosystems that could help mitigate salinity and drought stresses at the same time.

**Methods:**

The *Bacillus subtilis* ER-08 (BST) strain was isolated from the halotolerant plant Fagonia *cretica* which was collected from the Little Rann of Kachchh, India. Various biochemical and molecular approaches were applied for the detailed characterization of the BST isolate.

**Results and discussion:**

The BST isolate demonstrated notable plant growth-promoting qualities. Fenugreek seed biopriming was performed using the BST isolate. The effect of BST seed treatment on fenugreek developmental indices as well as abiotic alleviation was examined under greenhouse conditions. The BST produced 83.7 g ml^−1^ gibberellins (GA_3_) and 176.1 g ml^−1^ indole-3 acetic acid. Moreover, hydrogen cyanide, siderophore, exopolysaccharides (EPS), ammonia, cellulase, protease, pectinase, and chitinase were also produced by the BST strain. Interestingly, 52% of *Fusarium oxysporum* mycelial growth was suppressed by the BST isolate under *in vitro* conditions. Furthermore, BST isolates functioned well under several abiotic stress conditions, for instance, salinity (4 and 6 ds m^−1^), pH (5, 7, and 9), drought (PEG6000 at 10%, 20%, and 30%), and temperature (25°C, 35°C, 37°C, and 55°C). This study indicates that the BST strain might serve as an effective bio-inoculant for minimizing the detrimental effects of abiotic stresses.

## 1. Introduction

Fenugreek (*Trigonella foenum-graecum* L.) is placed under the Fabaceae family. Due to its wide adaptability, fenugreek is cultivated all over the world (Chaudhary et al., 2017). It is cultivated mainly in various parts of India like Gujarat, Rajasthan, Uttar Pradesh, Punjab, Madhya Pradesh, and Maharashtra (Nybe et al., 2007). Annually, in India, there is a production of ~90,000 metric tons of fenugreek on 66,000 cultivated hectares (Rani and Hegde, 2017). Fenugreek has exceptional nutritional value. The chemical components of fenugreek, which include neotigogenine, galactosamines, diosgenin, terpenoids, tigogenin, trigonellin, flavonoids, coline, isoleucine, and other phenolics, assure its numerous applications (Irankhah et al., 2021). In 2021, 203 thousand metric tons of fenugreek were produced in India [SRD (Statista Research Department), 2021]. However, fenugreek productivity, like that of many other crops, is negatively impacted by a variety of biotic and abiotic stressors.

Salinity stress has recently emerged as a major global agricultural issue, transforming ~20% of the total agricultural zone into uncultivable regions (Rasool et al., 2013), particularly arid and semiarid lands, at a projected annual rate of ~1%−2% (Mohanty et al., 2021). By 2050, salt pollutants will have an impact on nearly 50% of farming areas (Butcher et al., 2016). In India, salinity affects over 6.74 million hectares of land (Kumar and Sharma, 2020). The Indo-Gangetic region of India has the highest saline area coverage (Arora and Sharma, 2017). Multiple investigations have found that elevated salinity in agricultural soils has an impact on crop yield (Biswas and Biswas, 2014; Sahab et al., 2021; Upadhyay and Chauhan, 2022). According to a study carried out in northern India, fenugreek is more susceptible to excessive salinity than coriander and fennel plants (Yadav et al., 2013). A study conducted by Banakar and their team exhibited a decrease in the production of fenugreek plants along with increased soil salinity, i.e., by 10% (3.38 ds m^−1^), 25% (6.28 ds m^−1^), and 50% (11.67 ds m^−1^) (Banakar et al., 2022). One other major abiotic stress that negatively impacts the crop is drought, thereby affecting photosynthetic activity, nutrient uptake, and water association (Osakabe et al., 2014). The two main causes of drought in India are diverse physiographic scenarios and spatial variability in the southwest monsoon (Sam et al., 2020). India ranked second among Asian countries in terms of the intensity of drought incidents (Aleksandrova et al., 2014). Since 1990, India has undergone prolonged and substantial droughts in succession, and the frequency as well as the severity of these events are rising consistently (Adger, 2006). For over 115 years, billions of people in India have suffered and millions have perished as a result of drought-related catastrophes (Gandure et al., 2013). Along with the development and yield of crops, soil stability and characteristics are impacted by water deficiency circumstances. Furthermore, during a long-term drought, the amount of water available to leach the salts diminishes, potentially resulting in an excess of concentrated salt (Ma et al., 2020). Crop growth and development can be significantly impeded by the synergistic effects of salinity and drought (Mittler, 2006; Kaushal and Wani, 2016). Moreover, fenugreek production is also challenged by several diseases. *Fusarium* wilt is among the most prevalent diseases of the fenugreek plant and was first reported in the Jaipur district of Rajasthan, India (Shivpuri and Bansal, 1987). Increased yellowing, leaf defoliation, discolored roots, growth inhibition, and wilting of the entire plant are the characteristic symptoms of Fusarium wilt (Kumar et al., 2017). Fenugreek plants showing typical Fusarium wilt symptoms were reported in multiple locations in the Saurashtra region of Gujarat (Bhimani et al., 2018). Therefore, Fusarium wilt is causing significant damage to fenugreek production in India. Due to these reasons, it is necessary to switch to resource-efficient cultivating methods in order to ensure the sustainable production of fenugreek under stressful conditions (Upadhyay and Chauhan, 2022).

Seed bio-priming with beneficial rhizosphere microbes could serve as an effective technique for enhancing plants' resilience to adverse climatic conditions (Redondo-Gómez et al., 2022). Plant Growth Promoting Rhizobacteria (PGPRs) are able to adapt to varied environmental circumstances, which represents their potential as an eco-friendly substitute for stress alleviation (Nadeem et al., 2014; Vimal et al., 2017). Plants might be able to withstand a variety of stresses due to the capacity of PGPRs to trigger stress-adaptive biochemical and physiological stimuli (Hernández-Canseco et al., 2023; Kumawat et al., 2023). PGPR can have a direct or indirect impact on the growth and development of the plant (Chauhan and Upadhyay, 2023). A few of these processes include phytohormone biosynthesis, increased mineral nutrient solubilization, osmotic adjustment by reduced transpiration, activation of the antioxidant enzymes, nitrogen fixation, suppression of pathogens by siderophore development, antibiosis, and hydrogen cyanide (HCN) production (Islam et al., 2016; Ma et al., 2020; Patel et al., 2023; Ramasamy and Mahawar, 2023). In addition, some PGPRs have the capability to lower ethylene-induced damage by producing 1-aminocyclopropane-1-carboxylate (ACC) deaminase (Upadhyay et al., 2022a,b). By producing exopolysaccharides (EPS) and biofilms, PGPRs are extensively recognized for their ability to combat salinity and drought (Singh et al., 2022). Remarkably, PGPR-mediated stress alleviation processes operate sequentially or concurrently in an age-dependent pattern (Figueiredo et al., 2016). One of the most prominent PGPR taxa, *Bacillus*, could encourage the growth of plants by employing a bunch of strategies (Islam et al., 2016; Shafi et al., 2017; Sharf et al., 2021). Interestingly, previous investigations have shown that if rhizobacteria have the appropriate solute transport systems or the ability to synthesize them, they can develop salt tolerance through the accumulation of suitable solutes (Nagata et al., 2002). According to prior research, *Bacillus subtilis* can achieve salt tolerance through the accumulation of glutamic acid and K^+^ ions in the cytoplasm as their primary solute and ion, respectively (Ikeuchi et al., 2003). Therefore, detailed investigations are required to effectively employ multifunctional PGPR under field conditions and ensure sustained crop production.

It is noteworthy to mention that halophytic plants are a viable source of multi-trait halotolerant microbes, which can augment plant development and growth *via* diverse techniques (Etesami and Maheshwari, 2018). The halophyte-associated rhizobacteria could play a pivotal role in fostering plants' resilience to salinity (Kerbab et al., 2021; Patel et al., 2023). In light of this, authors speculate that halotolerant microorganisms from harsh environments might be used as bioinoculants for the long-term modulation of stress-mediated alternations of the plant's physiological responses. Consequently, the present investigation was conducted in order to discover and characterize PGPRs with varied PGP characteristics as well as to investigate the PGPR-mediated alterations of the physiological processes in fenugreek to combat drought and salinity.

## 2. Materials and methods

### 2.1. Sampling, isolation, and soil characterization

Rhizospheric soils (with complete root systems) of the *Fagonia cretica* plants grown in the Kachchh region of Gujarat, India (22°61′28^′′^N, 71°19′22^′′^E), were collected. The collected soils were kept in plastic bags in a refrigerator for further use. Rhizospheric soil, separated by gently agitating the roots to remove loosely adhering soil, was suspended in 100 ml of 1% NaCl solution and vortexed for 2–3 min. Further soil analysis was conducted according to the protocol described in our recently published manuscript (Patel et al., 2023).

### 2.2. Biochemical and molecular characterization of bacterial isolates

From the halotolerant plant, *F. cretica*, a substantial number of bacterial isolates were collected. A sequence of biochemical analyses was performed using “Bergey's Manual of Systematic Bacteriology” (Bergey et al., 1994). A total of 13 bacterial isolates were selected for PGP trait screening, and one of the 13 isolates was chosen for further investigation because this isolate (BST) demonstrated multiple plant growth promotional activities. Biochemical characterization of PGPR isolates was conducted according to Patel et al. (2023).

The isolation of bacterial genomic DNA was done by the “lysozyme-SDS-phenol/chloroform method” (Chen and Kuo, 1993). The 16S rRNA gene was amplified by polymerase chain reaction (PCR) using the bacteria-specific universal forward primers 27 F (5′-AGA GTT TGA TCC TGG CTC AG-3′) and reverse 1492 R (5′-AAG GAG GTG ATC CAG CCG CA-3′) under previously described standard parameters (Patel et al., 2023). The purification and sequencing of the PCR product of ~1,500 bp were done.

#### 2.2.1. Phylogenetic analysis

A homology search was conducted by utilizing the NCBI-BLAST search engine after the deposition of the sequences to the National Center for Biotechnology Information (NCBI). The sequences of the other reference strains (other members of the Bacillaceae family) and our group member (*Escherichia coli*) were obtained from the NCBI GenBank database. The alignment of sequences was done by applying Clustal X 2.0.11 and MEGA 11.0. Bootstrap replication provided statistical support for the phylogenetic tree nodes (1,000 replications). The Tamura-Nei model was used for the analyses (Tamura and Nei, 1993). The evolutionary analyses were performed with the help of MEGA11 (Tamura et al., 2021).

### 2.3. Plant growth-promoting characteristics of the rhizobacteria

#### 2.3.1. Phytohormones quantification

To investigate the production of indole-3-acetic acid (IAA) and gibberellic acid (GA_3_), high-performance thin-layer chromatography (HPTLC) was employed. BST was grown for 5 days at 28 ± 2°C in an Erlenmeyer flask containing Luria-Bertani (LB) broth (100 ml), to which tryptophan (2 mg ml^−1^) was added as a precursor of IAA. For Gibberellic acid (GA_3_) measurement, BST was cultured at 30°C for 5 days at 120 rpm in Jensen's broth medium. The technique for sample extraction and HPTLC quantification was done according to Patel et al. (2016).

#### 2.3.2. Solubilization of zinc, potassium, and phosphate

BST isolates were spot inoculated on Tris-minimal medium, Alexandrov agar medium (supplemented with 2% bromothymol blue), and NBRIP medium to confirm their ability to solubilize zinc, potassium, and phosphate, respectively. Medium composition (g L^−1^) was as follows: (a) Tris-minimal medium [Dextrose 10, (NH_4_)_2_SO_4_ 1, KCl 0.2, K_2_HPO_4_ 0.1, MgSO_4_ 0.2, pH 7.0, insoluble Zn compounds (ZnO) 0.1%, and Agar 15], (b) NBRIP medium [glucose 10, Ca_3_(PO_4_)_2_ 5, MgCl_2_.6H_2_O 5, MgSO_4_.7H_2_O 0.25, KCl 2, (NH_4_)_2_SO_4_ 0.1, and pH 7.0]. Alexandrov agar medium (ID: M1996) was purchased from HiMedia Laboratories Private Limited, Mumbai, Maharashtra, India. Bacteria culture was conducted according to the protocol described in our previous article (Patel et al., 2023). The positive response was suggested by the halo zone development surrounding the colony. The calculation of the solubilization index (SI) was done using the following formula:


$$\begin{array}{l} SI=Diameter\;\left(cm\right)+\;\frac{Halo\;zone\;\left(cm\right)}{Diameter\;\left(cm\right)} \end{array}$$

#### 2.3.3. Ammonia, siderophore, hydrogen cyanide, ACC deaminase enzyme, exopolysaccharides, and nitrogen fixation activity

BST isolates were cultured for 24 h at 28 ± 2°C in 1% peptone water inoculum, followed by the addition of 0.5 ml of Nessler's reagent. The formation of a yellowish-orange color is a sign that ammonia (NH_3_) is being produced. The BST was cultured on Chrome Azurol S (CAS) agar medium to assess siderophore production. The siderophore synthesis was identified by the establishment of a yellow-orange halo-zone around the isolated bacterial colonies against the control strain *Bacillus amyloliquefaciens* (GPB-2). GPB-2 was procured from the microbial stock of the departments of life sciences at HNGU. Approximately 4.4 g L^−1^ of glycine was added to King's B agar medium to assess the HCN production. Whatman Filter paper No. 1 was fixed to the lid of the Petri plate, followed by sealing with adhesive tape. The filter paper was then soaked with a mixture of Na_2_CO_3_ (2%) and picric acid (0.5%). When HCN was produced, the filter paper's color changed from yellow to orange, signaling a successful reaction. Dworkin and Foster (DF) minimum salts medium, having 3 mM ACC, was utilized for determining ACC deaminase activity (Dworkin and Foster, 1958). Composition of DF medium (g L^−1^): KH_2_PO_4_ 4, Na_2_HPO_4_ 6, MgSO_4_:7H_2_O 0.2, glucose 2, gluconic acid 2, and citric acid 2, with trace elements: 1 mg FeSO_4_:7H_2_O, 10 mg H_3_BO_3_, 11.19 mg MnSO_4_:H_2_O, 124.6 mg ZnSO_4_:7H_2_O, 78.22 mg CuSO_4_:5H_2_O, 10 mg MoO_3_, and pH 7.2. Bacteria were cultivated on a TSB medium and grown in a shaker incubator (120 rpm) for 72 h at 30°C to determine exopolysaccharides (EPS) formation (Verhoef et al., 2003). EPS was isolated by precipitation and dried at 58°C for 1 day in the same centrifuge tube to minimize errors, and the dry weight of EPS was determined. The isolate's capability to fix N_2_ was tested by maintaining it on Glucose Nitrogen Free Mineral Medium (GNFM medium) up to seven sub-culturings at 28 ± 2°C. The medium's green color turned blue after incubation, demonstrating the isolate's capacity to fix N_2_.

#### 2.3.4. Antioxidant activity assay

Approximately 3 ml of 0.1 mM 2-diphenyl-2-picryl hydrazyl hydrate (DPPH) was added to 1 ml of the BST supernatant followed by incubation in the dark for 30 min (Noha et al., 2021). The absorbance was measured at 515 nm in triplicate. Approximately 1 ml of ethanol was added to 3 ml of DPPH solution and used as a control. The following formula was used to compute DPPH scavenging activity:


$$\begin{array}{l} \text{DPPH radicals scavenging activity }\left(\%\right)=\\ \frac{\text{OD of the control}-\text{OD of the sample}}{\text{OD of the sample}}\times 100. \end{array}$$

#### 2.3.5. Hydrolytic enzyme (protease, chitinase, cellulase, amylase, and pectinase) production

Hydrolytic enzyme production by the BST isolate was done according to the previously described protocol (Patel et al., 2023). BST was spot inoculated and cultured at 30°C for 48 h in (a) skimmed milk agar media (for protease); (b) chitin agar media (for chitinase); (c) CMC (Carboxy Methyl Cellulose) agar media (for cellulase); (d) starch agar media (for amylase and starch breakdown); and (e) Pectinase Screening Agar Medium (PSAM) (for pectinase). The formation of a clear zone surrounding the bacterial colonies indicates a positive result.

#### 2.3.6. *In vitro* antimicrobial activity

The virulent *Fusarium oxysporum* isolate was acquired from the microbiological stock of the author's university department (Gujarat, India). For the bioassay, the pathogen and bacteria were co-cultured 3 cm apart on the same PDA (potato dextrose agar) plates and incubated for 7 days at 30°C (Alenezi et al., 2016). The calculation of the fungal growth inhibition was done using the following equation:


$$\begin{array}{l} Mycelial\;Inhibition\;\left(\%\right)=\left(1-\frac{a}{b}\right)\times 100 \end{array}$$

where “*a*” is the distance between the fungal growth edge (from the bacterial side) and the bacterial isolate growth edge (from the fungus side), and “*b*” is the distance between the fungal upper growth edge and the upper edge of the control petri dish.

#### 2.3.7. Endurance to abiotic stresses

The BST isolate was tested for its capacity to grow in nutrient agar media under a variety of conditions, including (a) different levels of sodium chloride (5%, 10%, 15%, and 20% NaCl), (b) varying pH values (5, 7, and 9), and (c) the ability to withstand drought stress [ability to grow at 10%, 20%, and 30% PEG 60000 (polyethylene glycol)]. For every test, 100 ml of bacterial culture media was prepared and cultured for 2 days at 30°C. Finally, bacterial growth was calculated using the optical density (OD) at 600 nm.

### 2.4. Root colonization

With a few minor modifications, the method proposed by Islam et al. (2016) was utilized to analyze bacterial isolates' root colonization. Briefly, plant roots were taken after the growth of 25, 35, and 45 days. Root structures were properly cleansed using tap water and then washed three times with SDW to remove adherent soil particles. Using a sterile mortar and pestle, 1 g of the sample was homogenized with 10 ml of SDW after the plant roots had been cleaned, blotted to dryness, and weighed. On PDA plates, serial dilutions were made, and the total number of CFU g^−1^ roots was measured after incubation for 24–48 h at 28 ± 2°C.

### 2.5. Seed bio-priming

For greenhouse studies, *T. foenum-graecum* seeds (physical purity: minimum 98%, genetic purity: minimum 95%, and germination: minimum 70%) were obtained from Dantiwada Agro Farm, Ahmedabad. Notably, 70% ethanol (for 1 min followed by washing three times with sterile distilled water) and 0.5% sodium hypochlorite (for 5 min followed by washing five times with sterile distilled water) were used to surface sterilize the seeds.

To prepare the bacterial inoculum, an overnight cultured bacterial suspension was added to nutrient broth and incubated at 28°C for 24 h in a shaking incubator at 120 rpm. After incubation, the culture was subjected to a centrifuge for the extraction of bacterial biomass, which was then suspended in distilled water. The optical density was measured at 600 nm and corrected to 0.1, corresponding to 10^7^ CFU ml^−1^. Surface-sterilized *T. foenum-graecum* seeds were immersed in bacterial cultures for 30 min. As a control, seeds were immersed in sterile distilled water.

The experimental soil was air-dried and filtrated using a sieve (2 mm), autoclaved, and then 5 kg were transferred into a polyethylene bag (25 cm in wide and 10 cm in height). Fenugreek seed sowing was carried out at a rate of 10 seeds per pot at a depth of 2–2.5 cm. Every day, sterile and deionized water was supplied to the pots to ensure optimum germination. The whole experiment was performed in an unregulated greenhouse under natural light and temperature conditions.

### 2.6. Pot experiments

The pot tests were conducted using a completely randomized block design to assess the ability of the multifaceted salt-tolerant bacteria to reduce the adverse effects of drought and salt stress in fenugreek plants. The experimental soil was obtained from the Hemchandracharya North Gujarat University's agriculture field (23°51′44.388^′′^N to 72°8′3.192^′′^E). The soil is classified as alluvial according to the Indian Council of Agricultural Research (ICAR), and its physicochemical properties are investigated. Plants were watered with distilled water without any salt in non-stressed conditions. According to Patel et al. (2010), soil salinity was measured under saline stress conditions (4 and 6 ds m^−1^).

The experiment to induce drought stress in fenugreek plants was performed in compliance with the protocol proposed by Batool et al. (2020). Briefly, the plant pots were watered every day with tap water at field capacity until the onset of the drought stress application period [30 days after sowing (DAS)]. Irrigation of pots was stopped for drought stress induction until soil relative water content (SRWC) reached 60% for moderate drought stress and 40% for severe drought stress. These water scarcities were retained for 7 days by daily monitoring of the soil moisture content and adjusting for the water loss. Conversely, at 80% SRWC, the control pots were properly hydrated. After 7 days of water-stressed conditions, all pots were properly re-watered until crop maturity (45 DAS). Before applying water to pots, the water status of the soil was evaluated using the following formula:


$$\begin{array}{l} \text{SRWC }\left(\%\right)=\text{FW}-\text{DW}/\text{TW }-\text{DW}\times 100 \end{array}$$

where DW indicates dry weight, FW indicates fresh weight, and TW indicates saturated soil weight assessed by saturating soil samples for 24 h.

### 2.7. Impact of BST seed bio-priming on the growth of fenugreek plants

Fenugreek plants were collected after 45 days of growth under drought and salt stress conditions, together with control conditions (without stress), and growth characteristics were determined. At the time of harvesting, the electrical conductivity of the soil suspension (1:5) of the individual treatment was measured in response to salinity stress. Seed germination (%) was measured at 10 DAS, and the percentage of seed germination was estimated using the following equation:


$$\begin{array}{l} \text{Seed germination }\left(\%\right)=\frac{\text{No}.\text{ of germinated seeds}}{\text{Total No}.\text{of sown seeds}}\times 100 \end{array}$$

A variety of morphological characteristics were investigated, including leaf area, shoot height, root length, root fresh and dry weights, and shoot fresh and dry weights. The vigor index was determined using the following equation:


$$\begin{array}{l} \text{Vigor index}=\text{Germination }\%\times \text{Total length of plant}. \end{array}$$

### 2.8. Biochemical and physiological analysis of fenugreek plants

The phenol sulfuric acid technique was used to determine the total soluble sugars (TSS) content of leaves, which was measured using the phenol sulfuric acid technique described by Krishnaveni et al. (1984). Glycine betaine was quantified using the methodology described by Grieve and Grattan (1983). The outcomes were recorded in millimoles of glycine betaine per kilogram of plant tissue water in the leaf and sample. The proline content of plant tissues was determined using the method proposed by Patel et al. (2014). The concentration of proline content was calculated using the following formula:


$$\begin{array}{r} \left(\text{ug proline in extract}/111.5\right)\;/\text{ g of sample }=\\ {\text{umol g}}^{-1}\text{of fresh tissue}. \end{array}$$

Approximately 1 g of plant tissue was chopped into tiny pieces and homogenized using a chilled mortar and pestle with 80% (V/V) acetone to determine the total chlorophyll concentration (Arnon, 1949). The total chlorophyll amount was presented as μg chlorophyll per gram of fresh tissue weight. The amounts of chlorophyll a and b were estimated using the following formulas:


$$\begin{array}{r} \text{Chlorophyll }“\text{a}\text{” }\left(\text{ug}/\text{ml}\right)\;=\left(12.7\;\times \text{OD at }663\text{ nm}\right)\\ -\left(2.69\;\times \text{OD at }645\text{ nm}\right)\\ \text{Chlorophyll }“\text{b}\text{” }\left(\text{ug}/\text{ml}\right)\;=\left(22.9\;\times \text{OD at }645\text{ nm}\right)\\ -\left(4.08\;\times \text{OD at }663\text{ nm}\right)\\ \text{Total chlorophyll }\left(\text{ug}/\text{ml}\right)\;=\left(20.2\;\times \text{OD at }645\text{ nm}\right)\\ +\left(8.02\;\times \text{OD at }663\text{ nm}\right) \end{array}$$

Total free amino acids and H_2_O_2_ content were measured using methods proposed by Sadasivam and Manickam (1996) and Loreto and Velikova (2001), respectively.

Lipid peroxidation was measured using the rate of malondialdehyde (MDA) production. One gram of fenugreek leaf tissues was mashed in 10% trichloroacetic acid (10 ml), and the mashed mixture was centrifuged for 20 min at 10,000 rpm. The reaction suspension, which comprised extract (2 ml) and thiobarbituric acid (2 ml), was heated for 30 min at 95°C, quickly cooled on ice, and afterward centrifuged at 10,000 rpm for another 20 min. The optical density of the supernatant was recorded using a spectrophotometer at 532 nm (A532), 600 nm (A600), and 450 nm (A450). The malondialdehyde concentration was determined using the following formula:


$$\begin{array}{l} \text{MDA content}=6.45\;\left(\text{A}532\;-\text{A}600\right)\;-0.56\text{ A}450 \end{array}$$

Membrane permeability was determined using the method and formula described by Lutts et al. (1996). The electrolyte leakage rate (ELR) is computed as follows:


$$\begin{array}{l} \text{ELR }\left(\%\right)=\left(\text{EC}2-\text{EC}1/\text{EC}3\right)\times 100 \end{array}$$

The relative water content (RWC) was measured using the method proposed by Teulat et al. (2003). The relative water content was determined using the following formula:


$$\begin{array}{l} \text{RWC }\left(\%\right)=\frac{\text{Fresh weight }-\text{Dry weight}}{\text{Fully turgid weight }-\text{Dry weight}}\times 100 \end{array}$$

The *T. foenum-graecum* plant's enzymatic antioxidants were measured according to the method described by Patel et al. (2014). Concisely, fresh leaf material (0.5 g) was crushed in 0.2 M cooled potassium phosphate buffer (5 ml). The homogenate was centrifuged for 20 min at 4°C at 10,000 *g*. Following that, the tissue extract was stored at −20°C for 48 h before being utilized to assess different antioxidant enzymes' activity such as superoxide dismutase (SOD), catalase (CAT), ascorbate peroxidase (APX), and glutathione reductase (GR).

Macro- and micronutrient contents were estimated according to Vaghela et al. (2010). The plant leaves were pounded with a mortar and pestle. The total nitrogen amount was calculated using the Kjeldahl technique, while the chlorostannous molybdophosphoric blue color method was used to measure phosphorus content. After triacid (HNO_3_:H_2_SO_4_:HClO_4_ in the ratio of 10:1:4) digestion, Mg, Ca, Zn, K, Fe, Mn, and Cu concentrations were evaluated by atomic absorption spectroscopy.

### 2.9. Statistical analysis

Throughout the study, the experiments were performed in triplicates. Furthermore, the mean value was taken as the final result with standard errors (SE). SPSS (version 25.0) was used for identifying the statistical differences between the treatments based on the DMRT tests (Islam et al., 2016) (level of significance *p* ≤ 0.05). The correlation plot was created using the “ggplot2 package” in the RStudio software (version R 4.2.3).

## 3. Results

### 3.1. Isolation of strains, biochemical characterization, and molecular identification

We examined the capacity of the BST isolate to support plant development and to tolerate biotic and abiotic stressors. Supplementary Table 1 displays the biochemical characteristics of the selected bacterial isolate, BST.

The BST isolate is an aerobic, gram-positive bacterium that can synthesize the enzymes catalase and extracellular proteolytic gelatinases. BST is also capable of utilizing citrate as a carbon source (Supplementary Table 1). Furthermore, as seen by the positive VP test result, BST can use the butylene glycol route to generate acetoin. However, less acid was produced by the BST isolate from the fermentation of glucose, as confirmed by the MR test (Supplementary Table 1). Except for maltose, mannitol, and xylose, BST isolate can ferment several sugar sources, including sucrose, lactose, dextrose, and fructose, as confirmed by the sugar fermentation test (Supplementary Table 1).

**Figure 1:**
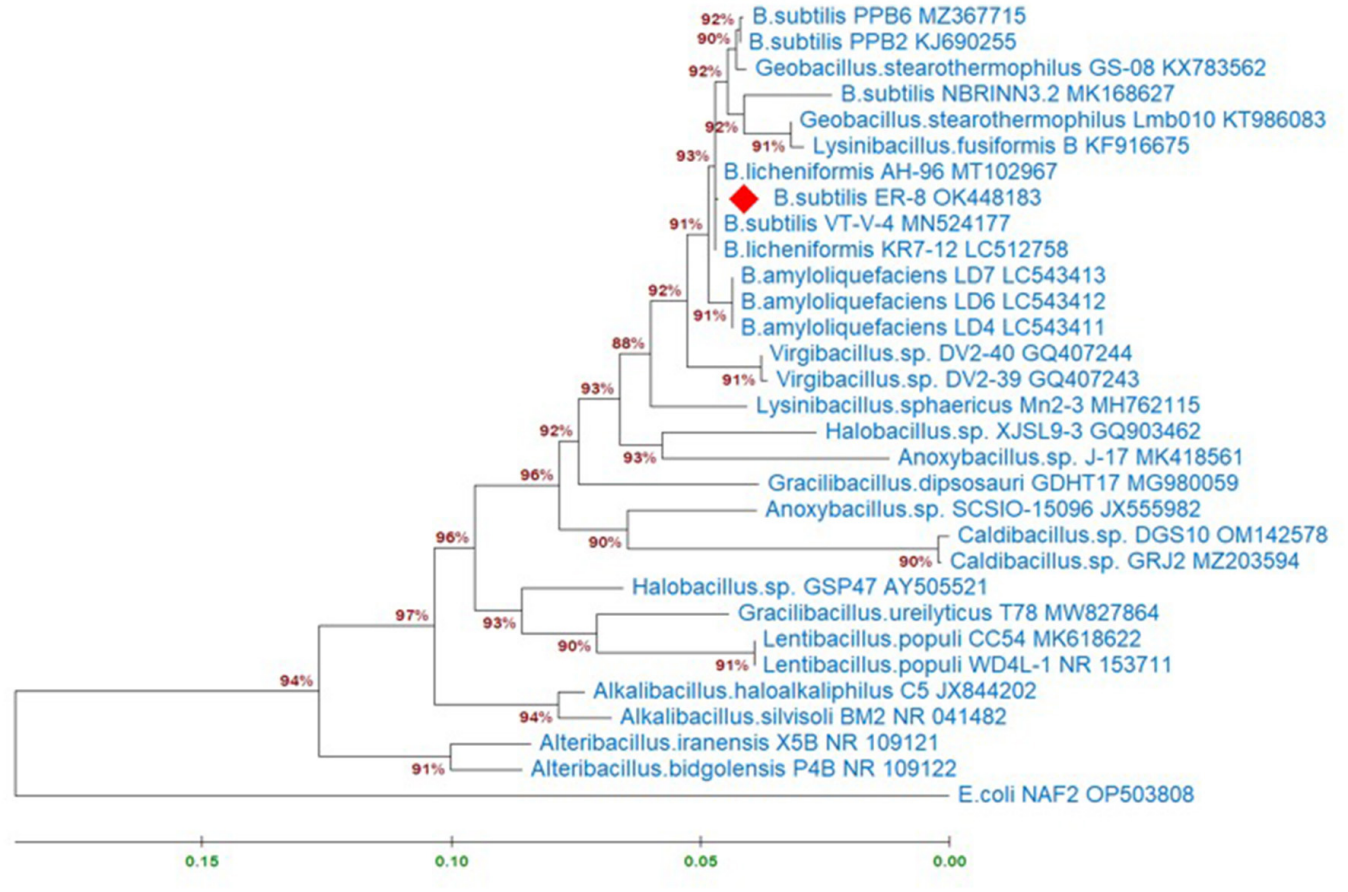
Maximum-likelihood tree displaying the relationships between the *Bacillus subtilis* ER-8 (BST) isolate and other genus of the Bacillaceae family based on the 16S rRNA gene sequences. Analyses were conducted using the Tamura-Nei model. This analysis involved 31 nucleotide sequences. There were a total of 1,479 positions in the final dataset. Evolutionary analyses were conducted in MEGA11. The information about related species was obtained from the NCBI GenBank database.

Molecular identification of the *B. subtilis* strain ER-08 (BST) was done by 16S rRNA gene sequencing. Figure 1 shows the maximum-likelihood tree created using the BST sequence (NCBI_accession # OK448183) and the reference sequences from the NCBI database. *Escherichia coli* was used as an outgroup member. A phylogenetic tree was prepared to find out the *B. subtilis* ER-08 (BST) strain's position within the other genus of the Bacillaceae family (Figure 1). The estimates of evolutionary divergence between these sequences expressed as ‘Patristic distances' between pairs of sequences are shown in Supplementary Table 2. The BST strain is closely linked to *Bacillus licheniformis* and *B. amyloliquefaciens*, showing only 0.08 and 0.70% patristic distances, respectively (Supplementary Table 2). However, the patristic distances of the BST strain from the rooted *E. coli* strain are 32.87% (Supplementary Table 2).

### 3.2. Physico-chemical assessment of soil

The rhizospheric soil of *F. cretica* belongs to the “sandy clay” soil-class, whereas the pot experimental soil was “silt.” Table 1 illustrates the physicochemical parameters of a rhizospheric soil sample and an experimental soil.

**Table 1:** Comparison of physicochemical properties of rhizospheric and pot experimental soil samples utilized in the current investigation.

**Soil parameters**	**Rhizospheric-soil sample**	**Pot experimental soil sample**
pH	7.80	7.40
EC (ds m^−1^)	5.1	0.62
Soil texture	Sand clay	Sand silt
OC (%)	0.44	0.75
N (%)	0.19	0.29
P (kg/ha)	15.2	45
K (kg/ha)	201	283
Ca (kg/ha)	229	167
Z (ppm)	8.27	19.5
Mn (ppm)	21.1	26.2
Cu (ppm)	6.38	10.6
Fe (ppm)	14.2	31.1

### 3.3. PGP traits of the BST isolate

**Figure 2:**
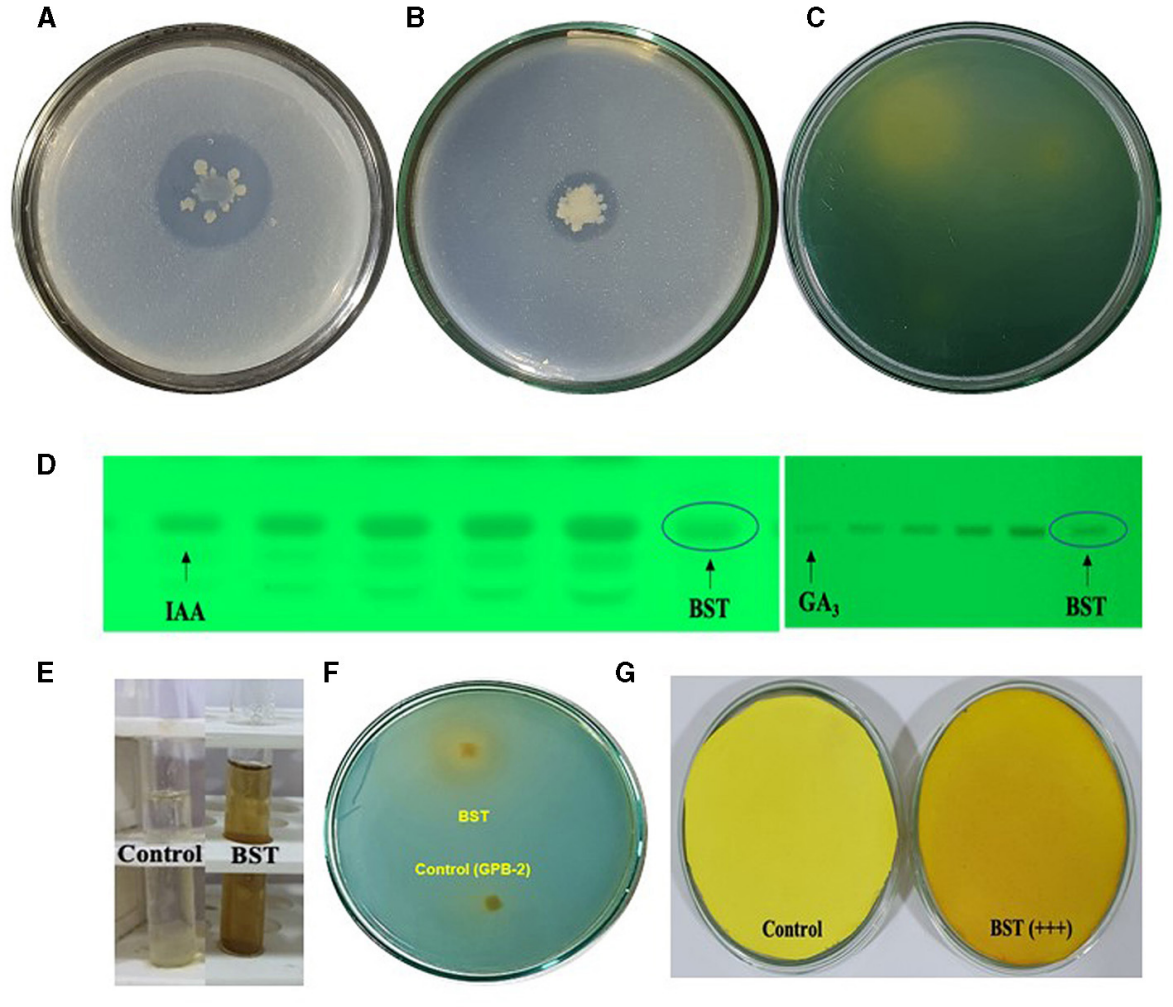
Plant growth-promoting attributes shown by the BST isolate in the *in vitro* condition. **(A)** P solubilization; **(B)** Zn solubilization; **(C)** K solubilization; **(D)** IAA and GA_3_ biosynthesis; **(E)** ammonia; **(F)** siderophore; **(G)** HCN synthesis.

The colony's halo zone provides proof of a positive test. For the solubilization of P, BST created a 28-mm halo zone, an 11-mm spot, and a 3.5-index (Figure 2A). Furthermore, for the solubilization of Zn, BST created a 17-mm halo zone, a 15-mm spot, and a 2.1 index (Figure 2B). Additionally, for solubilization of K, BST created a 32-mm halo zone, a 29-mm spot, and a 2.1 index (Figure 2C).

BST isolates generated IAA, GA_3_, ammonia, HCN, ACC deaminase, and EPS. The BST isolate's synthesis of IAA and GA_3_ was verified by HPTLC quantitation. BST was capable of producing 83.7 g ml^−1^ GA_3_ and 176.1 g ml^−1^ IAA (Figure 2D). In peptone water, BST produced 4.1 mol ml^−1^. The appearance of brown indicates that ammonia is being produced in large quantities (Figure 2E). A yellow-orange halo zone formed on a chrome azurol S (CAS) agar petri plate, confirming siderophore synthesis by the BST (Figure 2F). BST, on the other hand, was able to transform the Whatman filter paper no. 1 from yellow to dark brown, implying the formation of HCN. The BST-induced dark brown color shift (Figure 2G) demonstrates that HCN production is high (+++). Dowrking and Foster (DF) minimum salt agar plates amended with 3 mM ACC deaminase were used to cultivate strain BST. By catalyzing the only nitrogen source deamination process, the ACC deaminase synthesis by BST was assessed using the α-KB production technique. BST was able to produce 10.4 μmolh^−1^ mg^−1^ of α-KB protein ACC deaminase (Supplementary Table 1). Furthermore, BST was capable of producing exopolysaccharide (EPS). BST yielded 6.3 g L^−1^ dry weight of EPS (Supplementary Table 1).

### 3.4. *Bacillus subtilis* ER-08 (BST) has shown potential for disease suppressive ability through hydrolytic enzyme production and antagonistic activity

**Figure 3:**
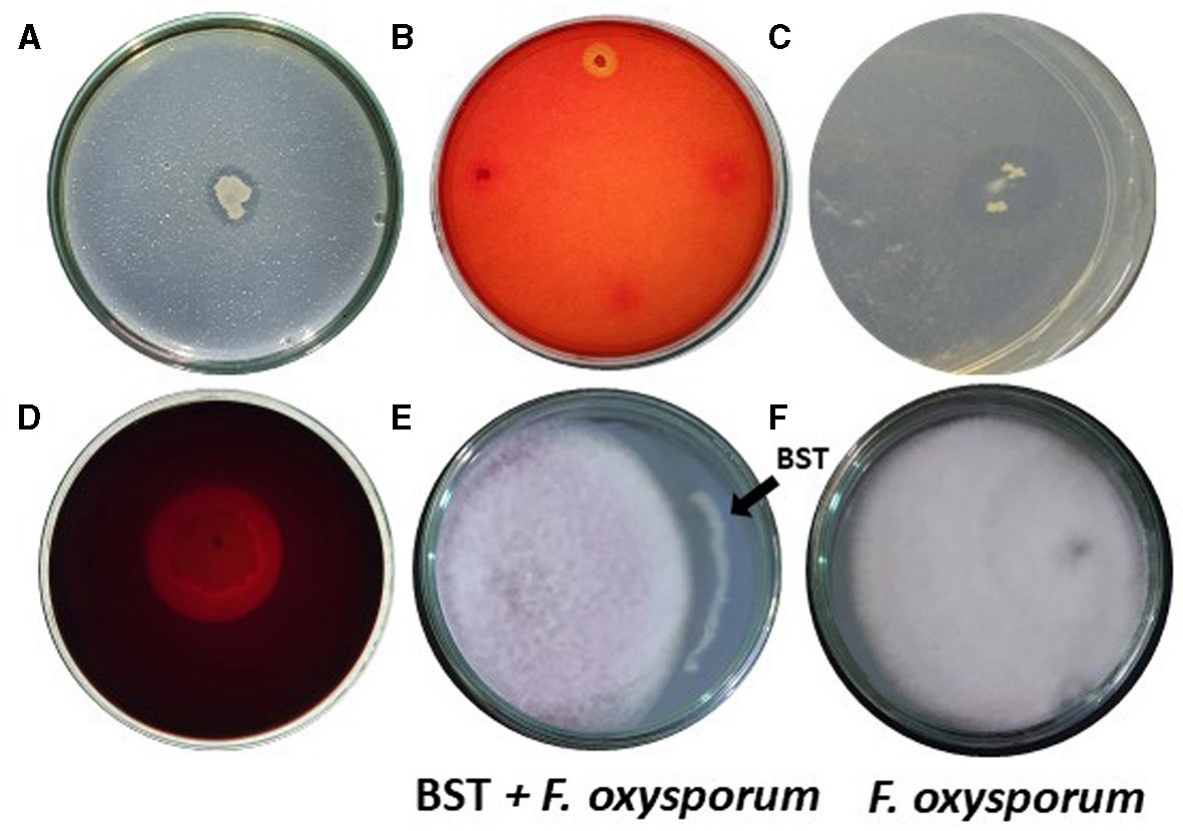
**(A–D)** Production of hydrolytic enzymes by strain BST. **(A)** Chitinase; **(B)** Cellulase; **(C)** Protease; **(D)** Pectinase; and **(E, F)** Mycelial growth inhibition of *F. oxysporum*.

We examined if isolated BST could generate hydrolytic enzymes. BST produced chitinase, cellulase, protease, and pectinase. On chitin agar plates, BST created a 17-mm halo zone of inhibition (Figure 3A). Furthermore, on CMC agar plates, BST created a 13-mm zone of inhibition (Figure 3B). On skimmed milk agar plates, BST suppressed protease activity by forming an 18-mm zone of inhibition (Figure 3C). On pectinase screening agar plates, BST was capable of producing a 29-mm halo zone of inhibition (Figure 3D).

Interestingly, BST has demonstrated potential for biological control of phytopathogens. BST inhibited the *F. oxysporum* mycelium's growth. Fifty-two percent growth inhibition was achieved, as shown in Figures 3E, F.

### 3.5. *Bacillus subtilis* ER-08 (BST) can sustain moderate to severe stress conditions

Abiotic stress tolerance of strain BST was investigated with 5%, 10%, 15%, and 20% salt concentrations; temperatures ranging from 25 to 55°C; pH ranges of 5.00, 7.00, and 9.00; and drought tolerance with 10%, 20%, and 30% polyethylene glycol (PEG) concentrations (Table 1). At 15% NaCl, 55°C temperature, and 30% PEG, strain BST endured drought stress. Isolate BST grew on alkaline medium with a pH of 9.00 and neutral media with a pH of 7.00, but not on pH 5.00. BST's capacity to thrive in NaCl concentrations as high as 15%, PEG concentrations as high as 30%, and temperatures as high as 55°C validated its halotolerant, drought-tolerant, and mild thermophilic nature (Supplementary Table 1). Therefore, the BST isolate has shown a high level of abiotic stress tolerance.

### 3.6. *Bacillus subtilis* ER-08 (BST) augments the growth of the fenugreek plants and increases plant nutrient elements under stressed conditions

**Figure 4:**
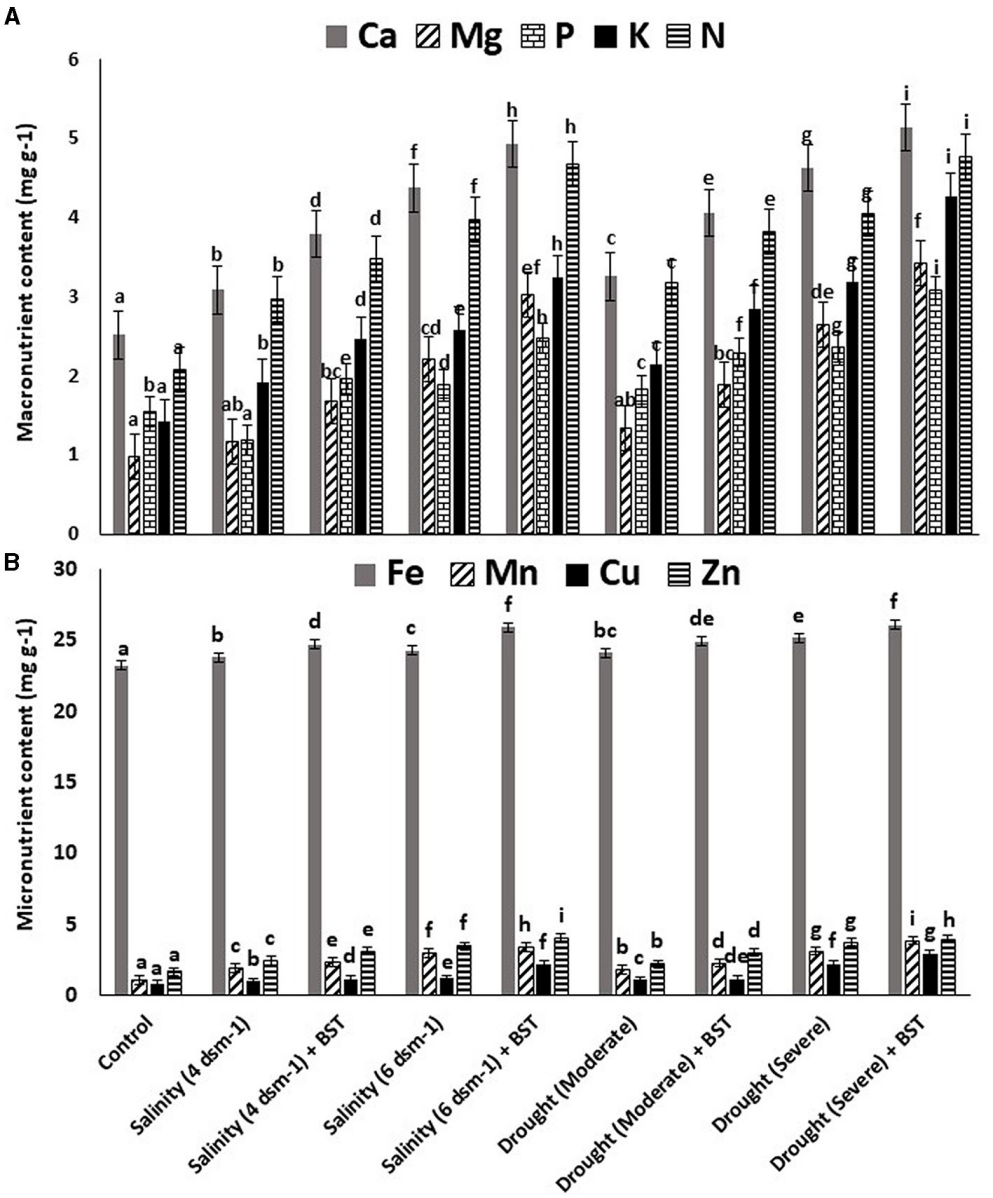
Effect strain BST inoculation on **(A)** macronutrient contents and **(B)** micronutrient contents. The error bars are the standard error (SE); the range is 0.2–0.4. The data signifies total mineral nutrient content (mg g^−1^), each from three sets of 8–10 samples. A statistically significant (*p* ≤ 0.05) difference between treatments is denoted by different letters inside each frame. At least two additional replications of the experiment were carried out.

The highest plant vigor, germination (%), and plant biomass enhancement were found under the treatment's salinity (4 ds m^−1^) + BST and drought (moderate) + BST conditions, which were statistically similar to non-treated control plants (Table 2 and Supplementary Figure 1). Significant (*p* ≤ 0.05) growth recovery was also observed under drought (severe) + BST and higher salinity stress [salinity (6 ds m^−1^) + BST] conditions (Table 2). As a consequence, the BST isolate has shown the efficiency to alleviate the harmful impacts of drought and salt stress on fenugreek plants. Furthermore, after seed treatment with the BST isolate, macronutrient (N, Ca, P, Mg, and K) and micronutrient (Mn, Fe, Zn, and Cu) concentrations in fenugreek plants improved significantly (Figures 4A, B).

**Table 2:** The plant growth metrics of the fenugreek plants accelerated by *Bacillus subtilis* ER-08 (BST) seed bio-priming.

**Plant growth parameters**	**Germination (%)**	**Shoot height (cm)**	**Root length (cm)**	**Shoot fresh weight (g)**	**Shoot dry weight (g)**	**Root fresh weight (g)**	**Root dry weight (g)**	**Vigor index**
Drought (severe)	30.00a	15.60a	4.50a	3.40a	0.50a	0.70a	0.20a	603.00a
Drought (moderate)	40.00b	18.90c	6.60c	4.50c	0.90c	1.00b	0.40b	1020.00c
Salinity (4 ds m^−1^)	50.00c	20.50d	7.30d	4.70c	1.10d	1.30c	0.70c	1390.00d
Salinity (6 ds m^−1^)	40.00b	17.40b	5.90b	3.90b	0.70b	0.80b	0.40b	932.00b
Drought (moderate) + BST	80.00f	22.30f	11.30g	5.40d	1.60e	2.40f	1.10d	2688.00g
Drought (severe) + BST	60.00d	20.80d	10.10e	4.60c	1.20d	1.80d	0.70c	1854.00e
Salinity (4 ds m^−1^) + BST	80.00f	23.50g	12.10i	5.90e	1.90f	2.90g	1.30e	2848.00i
Salinity (6 ds m^−1^) + BST	70.00e	21.20e	10.30f	5.20d	1.30d	2.10e	0.70c	2205.00f
Control	80.00f	22.10f	11.80h	6.10e	2.20g	2.80g	1.40e	2712.00h

A statistically significant (p ≤ 0.05) difference between treatments is denoted by different letters inside each frame. At least two additional replications of the experiment were conducted.

### 3.7. Effect of *B. subtilis* ER-08 (BST) on antioxidant enzyme activity

**Figure 5:**
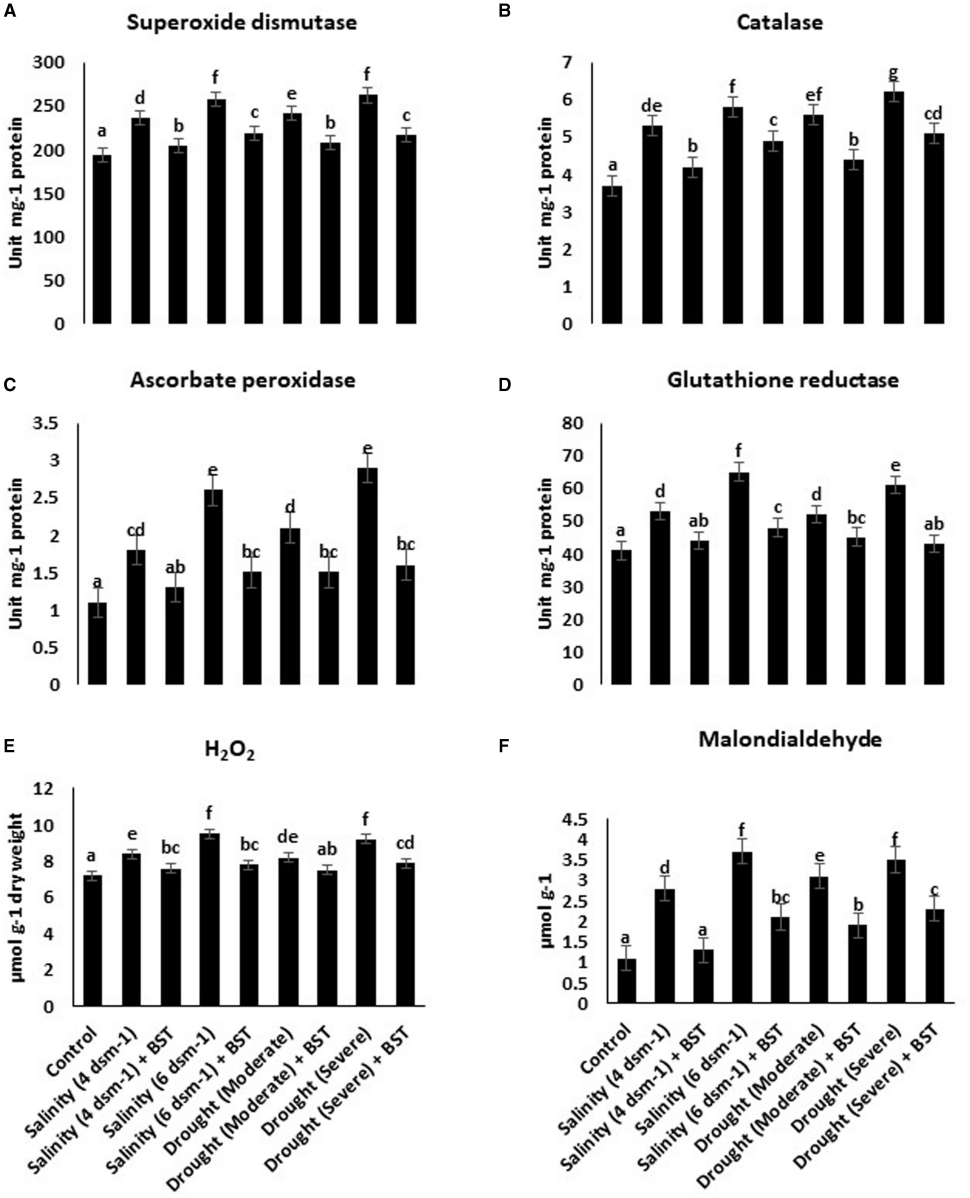
**(A–F)** *Bacillus subtilis* ER-08 (BST) inoculation positively regulates the AEA and reactive oxygen species (ROS) concentrations in fenugreek plants. The error bars are the standard error (SE). A statistically significant (*p* ≤ 0.05) difference between treatments is denoted by different letters inside each frame. At least two additional replications of the experiment were carried out.

The superoxide dismutase (SOD), glutathione reductase (GR), ascorbate peroxidase (APX), and catalase (CAT) activities were heightened under drought and salt stress conditions (Figures 5A–D). Conversely, antioxidant enzymes accumulation was reduced significantly following the BST isolate inoculation. BST inoculation also lowered the H_2_O_2_ and malondialdehyde concentrations (Figures 5E, F).

### 3.8. Substantial enhancement of the total free amino acid, chlorophyll, and total soluble sugar content followed by BST seed bio-priming

**Figure 6:**
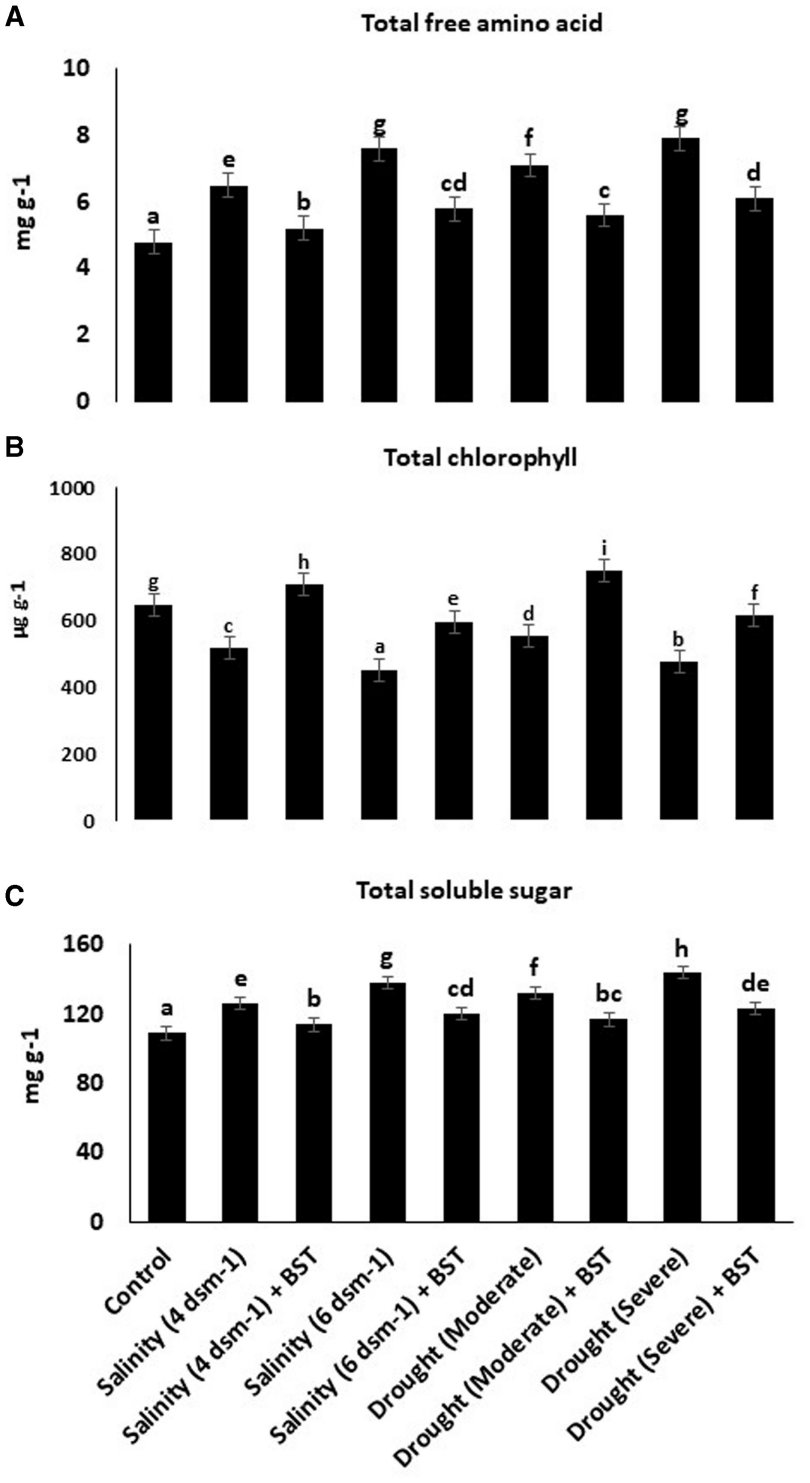
**(A–C)** Effect of *Bacillus subtilis* ER-08 (BST) on total free amino acid, total chlorophyll, and TSS content. The error bars are the standard error (SE). A statistically significant (*p* ≤ 0.05) difference between treatments is denoted by different letters inside each frame. At least two additional replications of the experiment were carried out.

Evidently, BST inoculation considerably increases the total free amino acids, chlorophyll, and TSS concentrations in fenugreek plants, as indicated by the data in Figure 6.

### 3.9. Reduction of the glycine betaine and proline content

**Figure 7:**
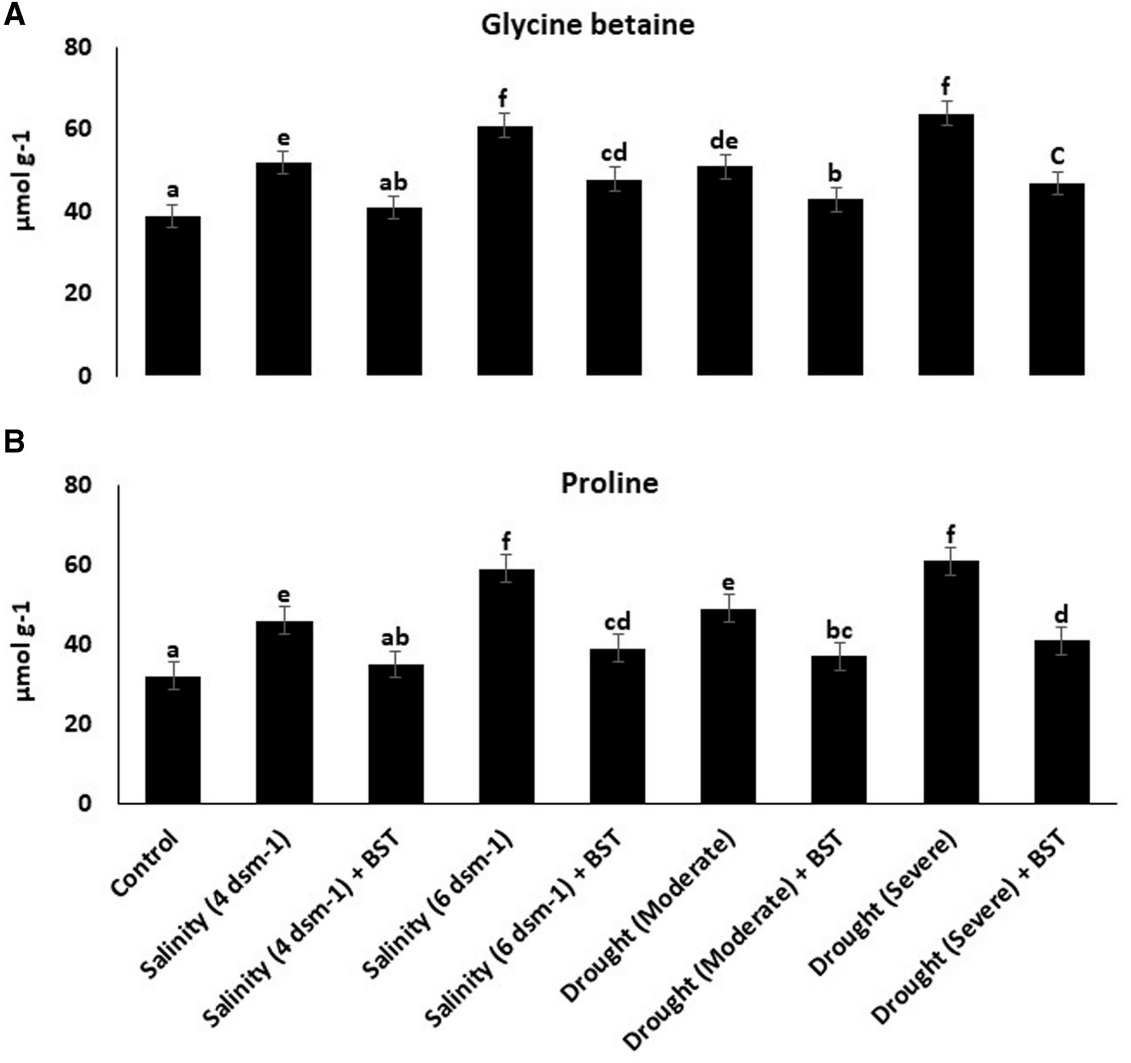
**(A, B)** Glycine betaine and proline content were lowered in BST-treated fenugreek plants, indicating the BST isolate's ability to generate tolerance against abiotic stressors. The error bars are the standard error (SE). A statistically significant (*p* ≤ 0.05) difference between treatments is denoted by different letters inside each frame. At least two additional replications of the experiment were carried out.

Following BST seed bio-priming, glycine betaine and proline concentrations in fenugreek plants decreased (Figure 7).

### 3.10. BST treatment reduced the occurrence of cell death and increased relative water content in fenugreek plants

**Figure 8:**
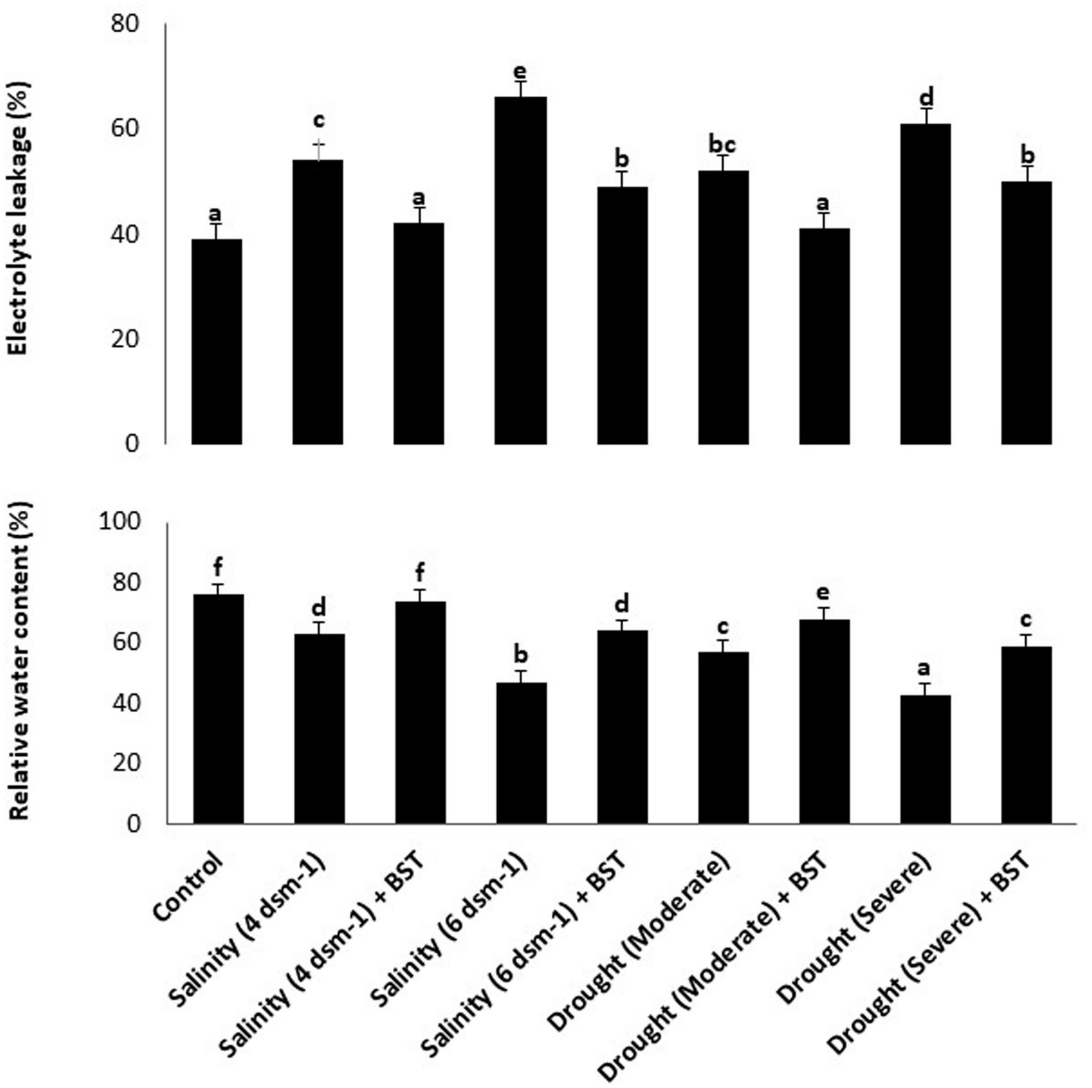
The impact of *Bacillus subtilis* ER-08 (BST) inoculation on the occurrence of cell mortality and RWC. The error bars are the standard error (SE). A statistically significant (*p* ≤ 0.05) difference between treatments is denoted by different letters inside each frame. At least two additional replications of the experiment were carried out.

According to findings from the electrolyte leakage assay, BST inoculation can considerably lower call mortality in plants that have been treated while also boosting the RWC of fenugreek plants (Figure 8). The highest cell death and lowest RWC were observed in the salinity (6 ds m^−1^) and drought (severe) treatments, respectively (Figure 8). The information presented here amply demonstrates the enormous potential of BST seed bio-priming to mitigate the negative impacts of salt and drought conditions.

### 3.11. DPPH radical scavenging activity

**Figure 9:**
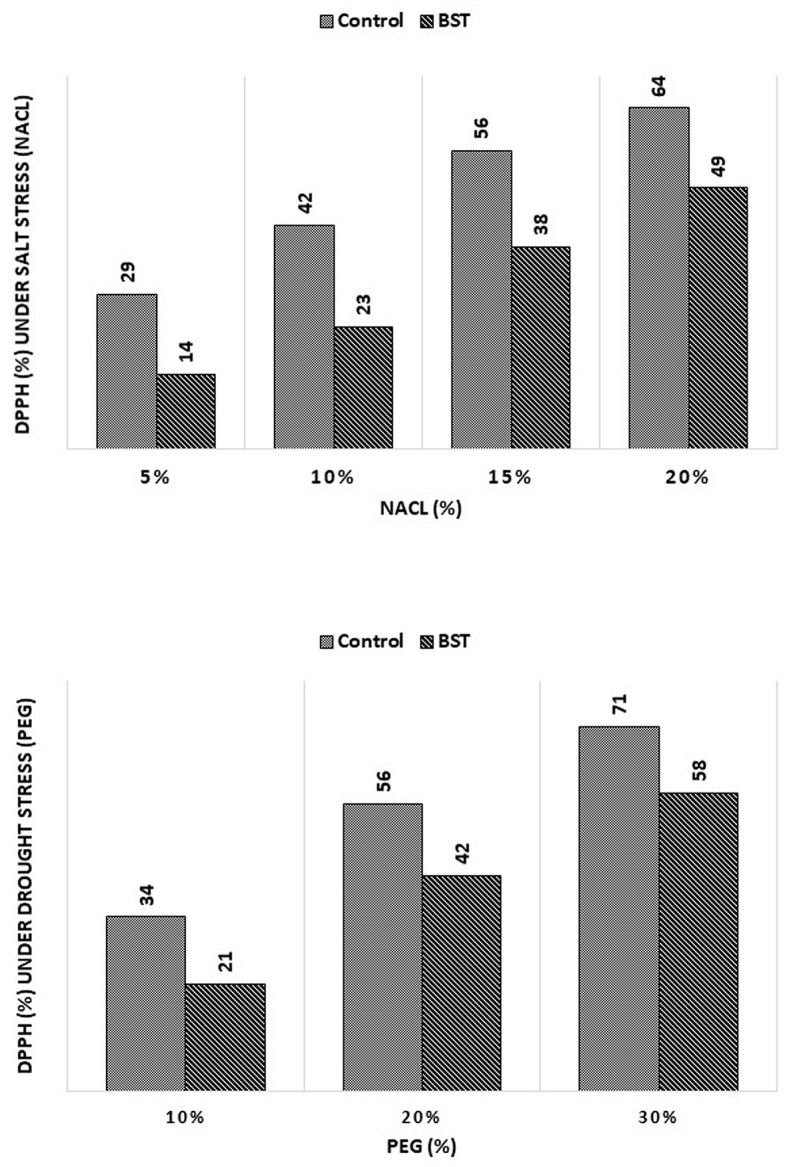
The effect of the *B. subtilis* ER-08 (BST) inoculation on DPPH radical scavenging activity. The experiment was carried out at least twice.

BST seed bio-priming considerably lessened the negative effects of the DPPH radical scavenging activity, as demonstrated in Figure 9.

### 3.12. Root colonization

**Figure 10:**
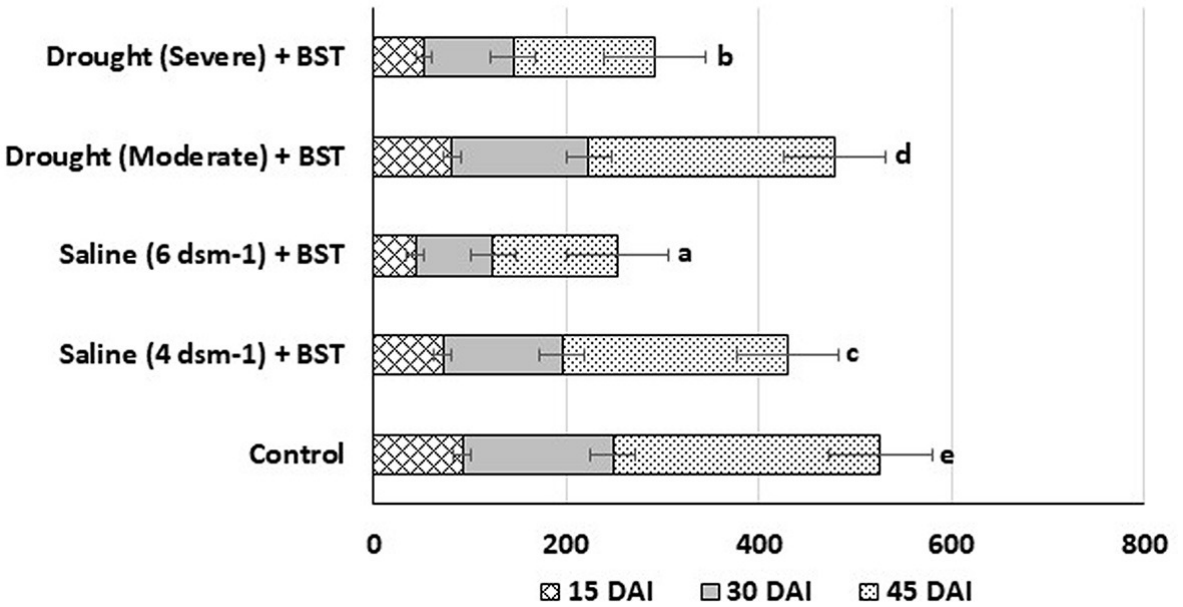
Population density (c.f.u.) of BST isolates at the 15, 30, and 45 DAI. The error bars are the standard error (SE). The data are provided as numbers of c.f.u. g^−1^ fresh weight, each from three sets of 5–8 whole roots. The data given are from exemplary experiments that were conducted twice and yielded similar findings each time. c.f.u., colony-forming units; DAI, days after inoculation.

To effectively stimulate plant development, rhizobacteria must be able to establish themselves within the root system. The *in planta* root colonization experiments showed the BST isolate colonized the test fenugreek plants efficiently within 15 days after inoculation (DAI). The colonizing bacterial populations were substantially increased on 45 DAI (Figure 10).

### 3.13. Correlation analysis

**Figure 11:**
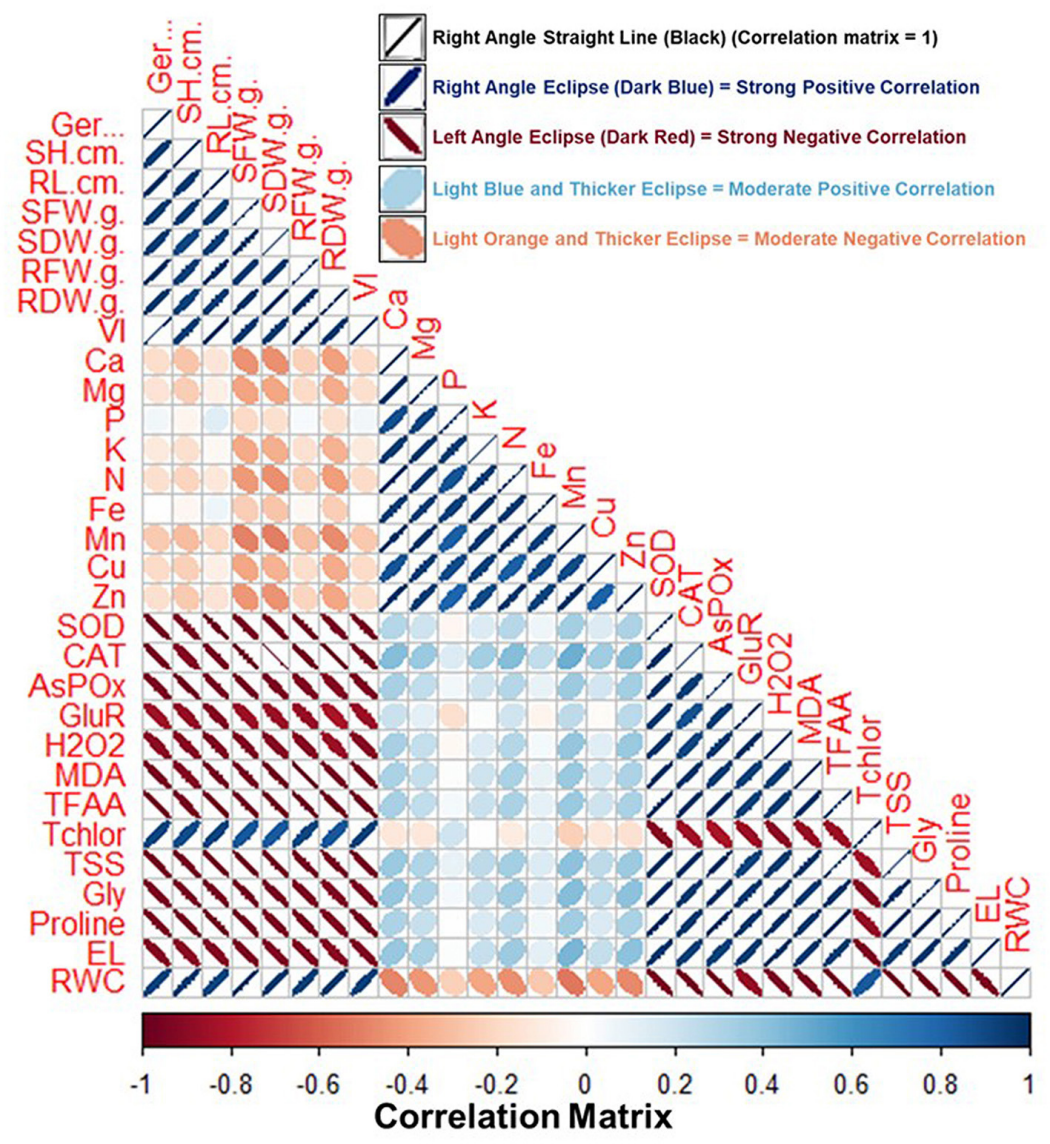
Correlation matrix of fenugreek plant characteristics impacted by the tripartite interactions among the BST seed bio-priming, salt, and drought stress. Here, Ger (%), Germination (%); RL, root length; RDW, root dry weight; RFW, root fresh weight; SH, shoot height; SFW, shoot fresh weight; SDW, shoot dry weight; Ca, calcium; VI, vigor index; Mg, magnesium; Cu, copper; Zn, zinc; SOD; CAT; APX; GR; H_2_O_2_, hydrogen peroxide; MDA, malondialdehyde; TFAA, total free amino acid; Tchlor, total chlorophyll; TSS, total soluble sugar; Gly, glycine betaine; EL, electrolyte leakage; RWC, relative water content. Right angle, dark blue color, and thin eclipse denote a strong positive correlation, while left angle, dark red color, and thick eclipse denote a strong negative correlation. The correlation matrix's values are shown in the “Legend” below the figure and included in the Supplementary Table 4.

Figure 11 shows the pairwise comparison between various growth and physiological parameters of the fenugreek plant influenced by BST inoculation. Pairwise comparison was done by correlation analysis between the various parameters. The information regarding the correlation matrix and the data that was utilized for preparing the correlation plot is shown in Supplementary Tables 3, 4. In the correlation plot, the right angle, dark blue color, and thin eclipse denote a strong positive correlation, while the left angle, dark red color, and thick eclipse denote a strong negative correlation (Figure 11). According to the correlation analysis, the different oxidative stress indicator metrics are adversely linked with plant biomass and growth parameters. In contrast, parameters related to plant biomass content, total chlorophyll, and RWC are positively correlated (Figure 11 and Supplementary Table 4). Thus, plant biomass and growth parameters are severely affected by the salt and drought-stressed conditions.

## 4. Discussion

The relationship between greater yields from agriculture and an increasing human population is complicated. As a result, feeding an increasing population that is projected to reach 10 billion by 2050 and caring for the environment is an intimidating task [GAP (Global Agricultural Productivity) Reports, 2018]. Global climate change factors like water scarcity and soil salinization are interlinked processes that adversely influence plant development, growth, and ultimately decrease crop production (Shelake et al., 2022). Excessive evaporation in semi-arid and arid areas may transport salt to the soil surface, creating saline-alkali soils (Hamal et al., 2021). As a result, crops cultivated in these regions are frequently exposed to both drought and salinity stress. The existence of these abiotic stress factors is a significant obstacle that has a negative effect on plants' physio-morphological and biochemical characteristics, impairing their ability to act normally and ultimately resulting in a drastic fall in agricultural yield (Shabbir et al., 2022). In light of this, it is critical to decrease and mitigate their negative impacts on crop development to secure agricultural sustainability and food safety.

Multifaceted halotolerant plant growth-promoting rhizobacteria (PGPR) can be the most promising approach for promoting plant growth and enhancing resilience against these abiotic stressors (Kumar et al., 2023). Consequently, changing environmental conditions have necessitated the discovery of safer, healthier, and more sustainable techniques for increasing crop yield. Employing microbes with multiple PGP properties could be one of the best techniques to induce tolerance against different abiotic stresses. Hence, the current study was undertaken to discover a novel multi-trait PGPR isolate and assess the effects of PGPR treatment on the fenugreek (*T. foenum-graecum* L.) plant under salt and drought-stressed conditions.

Our research showed that the *B. subtilis* ER-08 (BST) isolate from Gujarat, India's distinctive saline desert Little Rann of Kachchh, exhibited a variety of PGP characteristics as well as stress-relieving properties. The BST isolate was gram-positive, which was confirmed using the citrate utilization test. Citrate utilization is assumed to be crucial for bacterial persistence in roots and competing root colonization (Turnbull et al., 2001; Weisskopf et al., 2011). The BST strain was found to be positive for catalase and oxidase activity. The catalase test results of our BST strain support earlier research that found *B. subtilis* to be catalase-positive (Islam et al., 2016). The BST displayed multifarious plant growth-promoting (PGP) characteristics, including the ability to suppress the fungal plant pathogen *Fusarium oxysporum* and to tolerate different abiotic stresses such as pH, temperature, drought, and salt. The BST strain grew well on an alkaline medium with a pH of 9.00 and a neutral medium with a pH of 7.00. Furthermore, isolated BST was capable of withstanding salt (NaCl) concentrations of up to 15%, temperatures of up to 55°C, and PEG concentrations of up to 30%, suggesting halotolerant, drought-tolerant, and mild thermophilic traits. Inorganic potassium (K), phosphorous (P), and zinc (Zn) are solubilized by the BST isolate. BST's significant abiotic stress tolerance supports the close association between the origin of this strain and its tolerance abilities. Upadhyay et al. (2009) studied the genetic heterogeneity of halotolerant PGPR obtained from the wheat rhizosphere and revealed that most of the strains were capable of withstanding up to 8% NaCl concentration, which belongs to the *Bacillus* genus. pH and temperature were important factors in controlling the functionality and growth of microbes in the soil. Bacteria that exist in halophytes can endure NaCl concentrations ranging from 4 to 30% (Kerbab et al., 2021). These PGPBs (plant growth-promoting bacteria) are ideally suited for establishment in the plant rhizosphere because of their unique properties of competitiveness and persistence in saline and dry soil (Upadhyay and Chauhan, 2022; Upadhyay et al., 2022a,b).

Micro- and macronutrients are essential for the growth and development of plants. Unfortunately, due to diverse conditions such as saline conditions and prolonged drought stress, a large amount of soil nutrients may become inaccessible to the plants. Thus, nutrient-solubilizing rhizosphere microbial formulations are regarded as a viable solution to this particular problem (Rani et al., 2023). The crop plant benefits from bio-inoculation with PGPR because it makes insoluble (unusable) nutrients soluble (usable), increasing the nutrients' availability to the plants (Etesami and Glick, 2020). Interestingly, the BST isolate was able to solubilize P, Zn, and K. These qualities of enhancing the accessibility of inaccessible, insoluble nutrients are significant attributes of PGPR to improve the growth and production of crops (Danish and Zafar-ul-Hye, 2019; Kour et al., 2020).

The synthesis of GA_3_ and IAA is a frequent PGPR strategy to promote plant growth (Patten and Glick, 2002). IAA and GA_3_ increase the lateral roots, shoot height, and root length, which increase uptake of nutrients and improve plant health under both non-stressed and stressed conditions (Ullah et al., 2013). In the current investigation, the BST isolate generated a significant amount of both the GA_3_ and IAA phytohormones. Singh and Jha (2016) found that salt-tolerant *B. licheniformis* was able to solubilize phosphate, accumulate suitable solutes, and produce IAA and ACC, which may lessen salinity-triggered damage and increase plant tolerance under saline conditions. As IAA encourages the development of roots and the absorption of nutrients, it aids a crucial mechanism to stimulate plant growth (Carrillo et al., 2002). Thus, the increased nutrient content and enhanced ability to absorb nutrients in BST-inoculated fenugreek plants may be attributed to the influence of hormones on root architecture and activity.

PGPR-generated siderophore and HCN production have an indirect approach to action on plants as biocontrol agents (Kerbab et al., 2021). The siderophores are helping PGPR enhance the presence of essential nutrients for plants and also help plants absorb iron. Moreover, siderophores protect against pathogens and their harmful impact on the growth of plants (Zhou et al., 2017). Additionally, siderophore-producing bacteria improve all biochemical and physiological activities in plants exposed to several abiotic stressors (Kumar et al., 2016; Hofmann et al., 2021; Sultana et al., 2021). Ullah and Bano (2015) isolated the phosphate-solubilizing and siderophore-producing PGPR *Arthrobacter pascens* and *Bacillus* sp. from halophytes, which were found to be efficient in maize crop growth promotion under stressed conditions. One of the significant characteristics of PGPR is its ability to produce ammonia, a nitrogen source that indirectly and directly benefits crops (Richard et al., 2018). The ACC (1-aminocyclopropane-1-carboxylic acid) deaminase enzyme generated by halotolerant PGPRs is responsible for decreasing ethylene generation *via* transforming ACC (the plant-generated precursor of ethylene) into ammonia and α-KB (Yasmin et al., 2017; Etesami and Beattie, 2018). The ACC deaminase enzyme produced by PGPR provides protection to plants from the harmful impacts of ethylene under abiotic stress (Glick, 2014). Some PGPRs are able to produce EPS to defend themselves and their host plants against environmental variations and other abiotic stress factors such as salinity and drought (Upadhyay et al., 2011; Morcillo and Manzanera, 2021; Chauhan and Upadhyay, 2023). Moreover, PGPR lessens the influence of drought stress by modulating stress-responsive genes, changing root structure, increasing ACC deaminase enzyme activity, generating phytohormones, siderophores, osmolytes, exopolysaccharides, and volatile organic compounds (Ahmad et al., 2022). Multifarious halotolerant PGPR *K. variicola* SURYA6 excreted salt-mitigating metabolites such as EPS, ACC deaminase, IAA, and osmoprotectants that aid wheat and maize growth under salinity (Kusale et al., 2021). Mahmood et al. (2016) found that the EPS-producing salt-tolerant *Bacillus drentensis* and *Enterobacter cloacae* improved the growth of mung bean by increasing nutrient availability and water uptake in crop plants under salt stress. It's worth noting that the BST isolate considerably produced HCN, siderophore, ammonia, EPS, and the ACC deaminase enzyme. This demonstrates that BST isolate has a variety of properties that are advantageous to plants development and growth in adverse circumstances.

In addition to abiotic stresses, numerous soil-borne pathogens, such as different *Fusarium* species, pose a threat to fenugreek production in India (Bhimani et al., 2018; Ramteke et al., 2020). *Fusarium oxysporum* is an ascomycetous fungus that is responsible for numerous agriculturally important plant diseases. Results from our study indicate that the BST isolate, under *in vitro* conditions, can suppress the *F. oxysporum* pathogen. The ability to collapse fungal cell walls by producing hydrolytic enzymes is a characteristic of many biocontrol agents (BCAs) (Castillo et al., 2016). Cell wall-degrading enzymes are employed by BCAs to degrade plant cell walls so that they may be used as a primary carbon source (Khan et al., 2018). Previous research has demonstrated that the bacterial antagonists *B. subtilis* and *B. amyloliquifaciens* are effective BCAs (Dal Bello et al., 2002; Erlacher et al., 2014). According to Khan and colleagues, the 30VD-1 *B. subtilis* isolate produced a variety of antifungal compounds and volatiles to combat the plant pathogenic *Fusarium* spp. (Khan et al., 2018). Interestingly, the BST isolate can produce various hydrolytic enzymes, including chitinase, cellulase, protease, and pectinase, which could be attributed to its antifungal activity against *F. oxysporum*. As a result, additional *in planta* assessments of the biocontrol activities of the *B. subtilis* (BST) isolate will be conducted in the future. Moreover, the root colonization assay revealed that our BST isolate is a competent root colonizer, as CFU counts for the investigated strains exceeded 250 CFU g^−1^ root tissue. A previous study also tested root colonization by isolated bacteria under axenic conditions (Islam et al., 2016), which play a critical role for bacteria to survive inside plant roots (Turnbull et al., 2001).

Seed bio-priming with the BST isolate significantly increased seedling emergence and growth of fenugreek plants. The highest plant vigor, germination (%), and plant biomass enhancement were found in plants treated with BST as compared to control plants under salt and drought stress conditions. Plant biomass and seed germination are crucial indications of improved growth and development in plants (Tobe et al., 2005). Resilience against salinity at the stages of germination and seedling emergence defines improved plant establishment under salt stress (Bojovic et al., 2010; Keshavarizi and Mohammed, 2012). By reducing the amount of mycoflora that can inhibit a plant's capacity to survive, PGPRs can inadvertently boost the vigor index and seed germination (Begum et al., 2003). Naz et al. (2009) observed that the inoculation of the soybean plants with halotolerant bacteria resulted in enhanced dry biomass, shoot height, and root length because of GA_3_, IAA, and proline production. Therefore, BST seed bio-priming-mediated stimulation of fenugreek plant biomass and growth was validated by these earlier findings.

Photosynthesis is a vital plant biological process that retains plant development and increases tolerance against environmental challenges (Walters, 2005). Reduction in photosynthesis during salt stress frequently relates to declining chlorophyll pigment content (Gururani et al., 2015). Relative water content (RWC) indicates the water status of plants. Leaf RWC is the association between transpiration rates and water supply to leaf tissue (Lugojan and Ciulca, 2011). Enhancement of salinity in the root zone can cause a reduction in the water potential of the leaf and, thus, may affect several plant activities (Romero-Aranda et al., 2001). Interestingly, BST inoculation significantly increased the total chlorophyll content, RWC, macronutrients (Ca, Mg, P, N, and K), and micronutrients (Mn, Zn, Fe, and Cu) in fenugreek plants under drought and salt stress conditions. Plants treated with root-colonizing rhizobacteria increased the amount of chlorophyll pigment due to the reduced iron (Fe^3+^) in the siderophore-Fe^3+^ complex on the bacterial membrane to ensure iron availability to the bacteria and plant (Indiragandhi et al., 2008; Rajkumar et al., 2010).

Salinity-induced osmotic and ionic stress induces increased reactive oxygen species (ROS) generation, which causes oxidative damage to cells and eventually instigates the cell death response (Hasanuzzaman et al., 2021). Osmoprotectants such as glycine betaine, proline, and sugars assist in ion homeostasis to maintain cell turgidity and normal cellular character. Plants utilize a variety of antioxidant enzymes as part of their defensive strategy to disperse excessive ROS production and its harmful effects on the cell organs. When CAT is absent from the chloroplast, APX works with the GR to scavenge ROS and maintain redox equilibrium. The enzyme GPX detoxifies hydrogen peroxide (H_2_O_2_) and converts it into H_2_O. However, CAT degrades H_2_O_2_ by forming H_2_O and O_2_. As a result, CAT and GPX play key roles in the ROS detoxification pathway (Hasanuzzaman et al., 2020). The PGPR produced antioxidant enzymes are contributing to salinity stress tolerance in plants by decreasing H_2_O_2_ content (Li et al., 2020). Lipid peroxidation is measured by malondialdehyde (MDA) accumulation and has been utilized as an efficient standard for defining the sensitivity of plants to salinity (Ashraf and Ali, 2010; Ahmad et al., 2014). In this study, BST-treated plants showed enhanced concentrations of soluble sugars and total free amino acids while reducing electrolyte leakage when compared to uninoculated plants. In comparison to untreated plants under drought and salt stress conditions, BST treatment reduced the buildup of H_2_O_2_, MDA, glycine betaine, proline, and antioxidative enzymes such as glutathione reductase (GR), superoxide dismutase (SOD), ascorbate peroxidase (APX), and catalase (CAT). When introduced to salt and drought stress, plants inoculated with PGPR have a better capability to scavenge ROS due to higher MDA and H_2_O_2_ concentrations than plants without PGPR treatment. Tomato plants were cultivated with halotolerant PGPRs under salt stress conditions, resulting in enhanced chlorophyll, proline, and total soluble sugar content (Patani et al., 2023). Vardharajula et al. (2011) observed that drought-tolerant PGPR *Bacillus* sp. produced antioxidants and osmolytes that aid maize crop growth under drought stress. The PGPR *B. subtilis* HAS31-treated potato plants maintained higher dry biomass, soluble proteins, chlorophyll, total soluble sugars, and decreased ROS and MDA production under drought stress (Batool et al., 2020). The study demonstrated a reduction of antioxidant enzymes such as APX, GR, and CAT when wheat crop plants were inoculated with phosphate solubilizing strains of *B. subtilis* and *Arthrobacter* sp. under stressed circumstances (Upadhyay et al., 2012). Our research showed a parallel tendency in the BST seed bio-priming of fenugreek plants.

Ultimately, the BST isolate demonstrated various plant growth-boosting features as well as antifungal and abiotic stress alleviation properties. Different biochemical and metabolomics analyses have demonstrated that the BST isolate helps fenugreek plants in various modes of action, comprising protection against salt and drought stress, competitive root colonization, antagonistic activity against phytopathogens, and plant growth stimulation. It demonstrates the BST strain's enormous potential for employing it in the field as a biocontrol agent in addition to a biofertilizer. However, getting from evaluating PGPR potential to employing biofertilizers takes a long time, involving greenhouse studies with different types of soil in pots and subsequently field tests to determine the suitable inoculum compositions. The subsequent research will concentrate on the real-world application of robust integration of bioformulations to ensure efficient implementation of biological management strategies.

## 5. Conclusion

Global agricultural productivity has been impacted by climate change's escalating harshness of environmental stresses during the present Anthropocene period. In addition, agricultural productivity must be increased to ensure food safety for a globally increasing population while simultaneously creating more sustainable agriculture. From the halotolerant plant *F. cretica*, we have identified the stress-resilient, multifunctional plant growth-promoting rhizobacterial isolate *B. subtilis* ER-08 (BST). Fenugreek (*T. foenum-graecum* L.) growth under salt and drought stress was also shown to be promoted by this strain, which likewise exhibited various traits that promote plant growth. The findings of this study clearly demonstrate that the multi-trait strain, by supporting the entire plant at physiological and biochemical levels, plays a significant role in improving plant growth under abiotic stress. Under a variety of climatic conditions, these multitasking-beneficial bacteria are essential for eco-friendly agricultural operations and have the capacity to produce bacterial inoculants that operate as dual-purpose biostimulants.

## Data availability statement

The raw data supporting the conclusions of this article will be made available by the authors, without undue reservation.

## Author contributions

Conceptualization and supervision: AP, SB, B-HJ, and MJ. Investigation and methodology: MP, VY, FH, and KY. Original draft preparation: MP, SI, and AP. Review and final editing: MP, SI, H-KP, VY, and AP. All authors contributed to the article and approved the submitted version.
