# Supplementary material for: Bacillus subtilis ER-08, a multifunctional plant growth-promoting rhizobacterium, promotes the growth of fenugreek (Trigonella foenum-graecum L.) plants under salt and drought stress
Source: Front Microbiol. 2023 Aug 24;14:1208743. doi: 10.3389/fmicb.2023.1208743 (PMC10483830; doi:10.3389/fmicb.2023.1208743)
Supplement: Supplementary file 1 [file Table_1.docx]

**Supplementary Table 1** Biochemical characterization and stress tolerance of the *B. subtilis* ER-08 isolate

| **Biochemical Assays** | **Test Results** |
| --- | --- |
| Gram’s staining | Positive |
| Voges-Proskauer (VP) test | Positive |
| Catalase test | Positive |
| Nitrate reduction | Negative |
| Gelatin hydrolysis | Positive |
| Urea hydrolysis | Negative |
| Casein hydrolysis | Positive |
| Methyl Red (MR) test | Negative |
| Oxidase test | Positive |
| Citrate utilization | Positive |
| ACC deaminase activity | 10.4 μmolh^-1^mg^-1^ of α-ketobutyrate (α-KB) protein |
| EPS production | 6.3 g L^-1^ (dry weight) |
| **Sugar fermentation test** | |
| Maltose | Negative |
| Sucrose | Positive |
| Lactose | Positive |
| Dextrose | Positive |
| Fructose | Positive |
| Mannitol | Negative |
| Xylose | Negative |
| **Growth of the BST at various pH level** | |
| **pH** | **Growth of the BST isolate** |
| 5.00 | Negative |
| 7.00 | Positive |
| 9.00 | Positive |
| **Growth of the BST at different temperature level** | |
| **Temperature (°C)** | **Growth of the BST isolate** |
| 25 | Positive |
| 35 | Positive |
| 45 | Positive |
| 55 | Positive |
| **Growth of the BST at different NaCl concentrations** | |
| **NaCl (%)** | **Growth of the BST isolate** |
| 5 | Positive |
| 10 | Positive |
| 15 | Positive |
| 20 | Negative |
| **Growth of the BST at different PEG concentrations** | |
| **PEG (%)** | **Growth of the BST isolate** |
| 10 | Positive |
| 20 | Positive |
| 30 | Positive |
