# Supplementary figures and images for: Bacillus subtilis ER-08, a multifunctional plant growth-promoting rhizobacterium, promotes the growth of fenugreek (Trigonella foenum-graecum L.) plants under salt and drought stress
Source: Front Microbiol. 2023 Aug 24;14:1208743. doi: 10.3389/fmicb.2023.1208743 (PMC10483830; doi:10.3389/fmicb.2023.1208743)

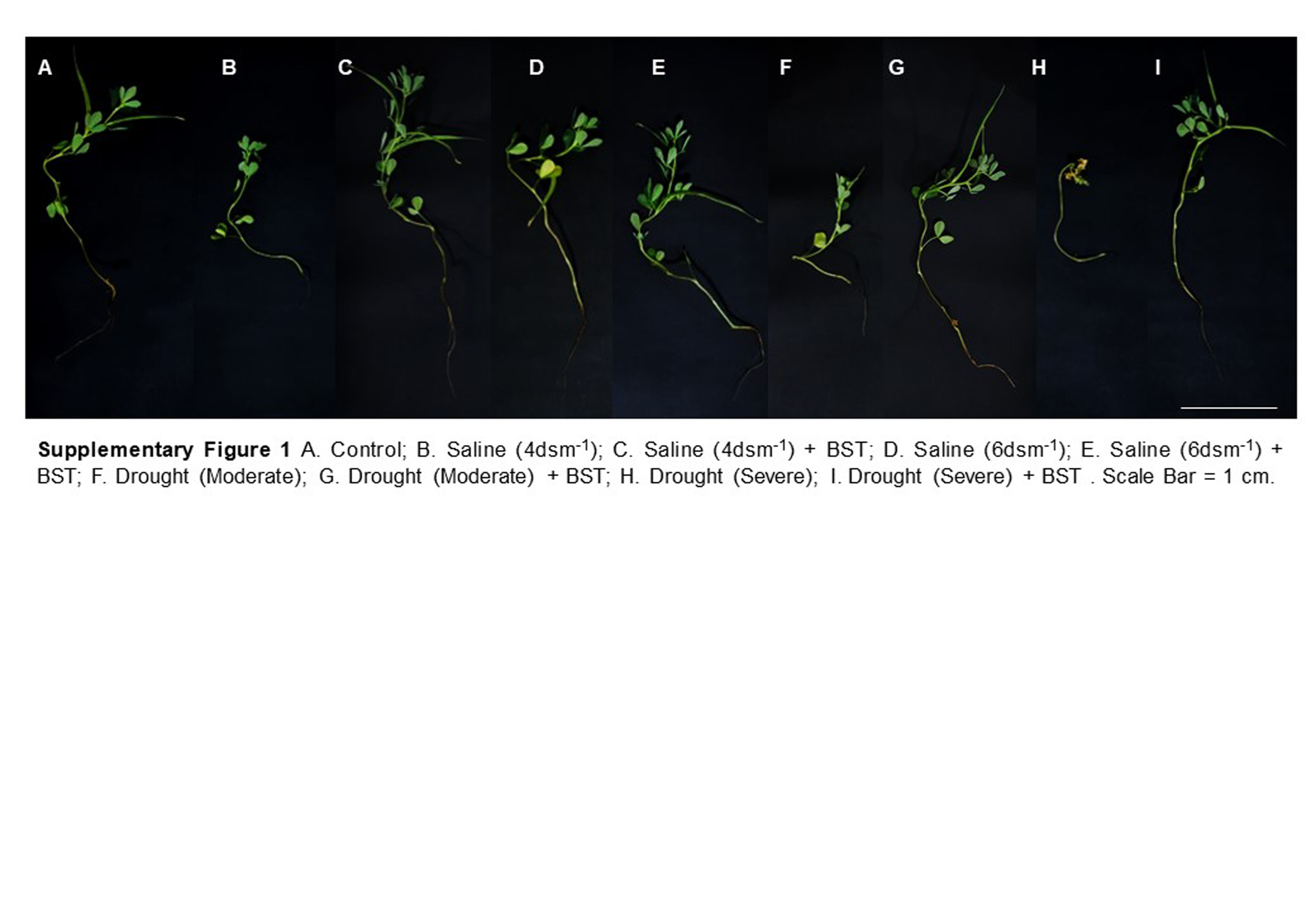

Supplement: Supplementary file 5 [file Image_1.JPEG]
